# A Scoping Review on the Association between Night Eating Syndrome and Physical Health, Health-Related Quality of Life, Sleep and Weight Status in Adults

**DOI:** 10.3390/nu15122791

**Published:** 2023-06-18

**Authors:** Sai Janani Sakthivel, Phillipa Hay, Haider Mannan

**Affiliations:** 1 Translational Health Research Institute, School of Medicine, Western Sydney University, Campbelltown, NSW 2560, Australia; 19488312@student.westernsydney.edu.au (S.J.S.); p.hay@westernsydney.edu.au (P.H.); 2 Mental Health Services SWSLHD, Campbelltown, NSW 2560, Australia

**Keywords:** night eating syndrome, obesity, sleep, physical activity, obstructive sleep apnoea, diabetes mellitus, cardiovascular disease

## Abstract

Night eating syndrome (NES) is characterised by recurrent episodes of night eating, evident through excessive food consumption after the evening meal or eating after awakening from sleep, often associated with significant distress and/or impairment in functioning. This scoping review was conducted according to PRISMA-ScR (Preferred Reporting Items for Systematic Reviews and Meta-Analyses for Scoping Reviews) guidelines. The search was conducted through the use of PubMed, Medline (OVID) and SCOPUS, to identify relevant articles published within the last 10 years. Search terms including “Night eating*” OR “NES” and Boolean phrases were used to refine the search. Additionally, the age of participants was restricted to 18 years and above, to ensure only adult participants were included. The abstracts of the remaining articles were used to screen for those that were relevant. From a total of 663 citations, 30 studies assessing night eating syndrome met the inclusion criteria to be included in the review. We found inconsistent associations of NES with higher body mass index (BMI), less physical activity, type 2 diabetes mellitus (T2DM) and poorer quality of sleep. These inconsistencies may have been due to the use of different measurement methods, lack of power from small sample sizes of NES in some studies and varying ages of participants, with associations being more likely to be found in higher-quality, representative populations than in university student samples. There were no associations of NES with T2DM in clinical populations and with hypertension, OSA and metabolic syndrome, but sample sizes were small. The impacts of NES on these medical conditions should be addressed in future, using well-sized and long-term studies involving representative populations of adults. In conclusion, NES likely has negative impacts on BMI, T2DM, physical activity, and sleep quality, which in turn may increase cardio-metabolic risk. However, further research is needed to elucidate the interaction between NES and its associated features.

## 1. Introduction

Night eating syndrome (NES) is an eating disorder characterised by recurrent episodes of night eating evident through excess consumption of food after the evening meal or nocturnal ingestions after awakening from sleep, which cause significant dysfunction and distress [1,2]. Although the aetiology of NES is poorly understood, the syndrome is thought to result from a desynchronisation of mood, sleep, satiety, and circadian rhythms of food ingestion [2,3]. Moreover, NES has a substantial association with concurrent psychiatric diagnoses and comorbidities; these can include binge eating disorder, bulimia nervosa, generalised anxiety disorder and substance use disorder [4]. Consequently, individuals with NES often experience significant distress and impairments in normal functioning [1,2,3]. With a prevalence of 1.5% in the general population of the United States [3], NES creates a substantial burden to the healthcare system, detrimentally impacting quality of life and increasing morbidity [2,3,4,5]. Whilst its morbidity is suspected to be moderate due to known associations with medical and psychiatric conditions, we found no specific statistics regarding NES and mortality. 

In addition to its mental health impacts, people with NES may also experience comorbidities such as sleep disorders and sleep apnoea, and metabolic conditions such as diabetes or hypercholesterolemia [2,4]. These problems are known to increase cardio-vascular and metabolic risk in the adult population and are associated with significant mortality and morbidity [4]. As with other EDs characterised by episodes of excess consumption, such as binge eating disorder [1], NES is also associated with increased body weight, and this further adds to the risk of metabolic syndrome and other physical health problems associated with obesity [2,4]. 

Research to date regarding the association between NES and Body Mass Index (BMI; kg/m^2^), physical health and sleep conditions is inconsistent [6,7,8]. Additionally, the majority of research on NES has been conducted within clinical settings [9,10,11] or in adolescent populations [12,13]. As such, there is a lack of knowledge regarding NES and physical health problems within more general and adult population groups [9,14,15]. To our knowledge, however, there has not been a scoping review or systematic review investigating the association of these physical health problems with NES. Thus, this review adopted a scoping review methodology to broadly investigate the extant literature, assessing associations of NES with physical health conditions, physical activity, sleep, and weight status in adult population samples of 18 years of age or older. We elected to conduct a scoping review as our research question was broad and we planned to optimise the inclusivity of articles to determine future directions in empirical and review research methodologies. This review searched articles from the past decade in order to focus on contemporary and up-to-date knowledge.

## 2. Materials and Methods

### 2.1. Search Processes 

This scoping review was conducted according to PRISMA-ScR (Preferred Reporting Items for Systematic Reviews and Meta-Analyses-Scoping Review) guidelines [16]. The search was conducted through the use of PubMed, Medline (OVID) and SCOPUS to identify relevant articles published within the last 10 years (until 2013). Search terms including “Night eating* OR “NES” and Boolean phrases were used to refine the search. Additionally, the age of participants was restricted to 18 years and above, to ensure only adult participants were included. The abstracts and titles of the remaining articles were then used to screen for articles that were of relevance. Additionally, all articles selected by title and abstract were read to ensure relevance to NES. 

### 2.2. Study Selection 

Articles were included in this scoping review according to the following inclusion criteria: published in the English language, reported on participants with NES who were over the age of 18 years, published in peer-reviewed journals and published in the last decade (since 2013). Articles were excluded if the full text was not available. References from systematic reviews and meta-analyses were also examined to identify additional original studies. We chose to review the impacts of NES on adult populations as a large proportion of the existing literature has been conducted on adolescent populations [12,13]. Articles were then screened based on title, abstract or full text due to irrelevance to topic question or where text was not fully available. 

### 2.3. Data Extraction 

After reviewing the results of the initial search based on inclusion and exclusion criteria, data were extracted and compiled into table format. Data included author names, publication dates, study designs, sample size, participant demographics and outcomes. Two authors (SS and PH) reached consensus on data that were extracted and included in these tables.

## 3. Results

### 3.1. Study Characteristics 

**Figure 1:**
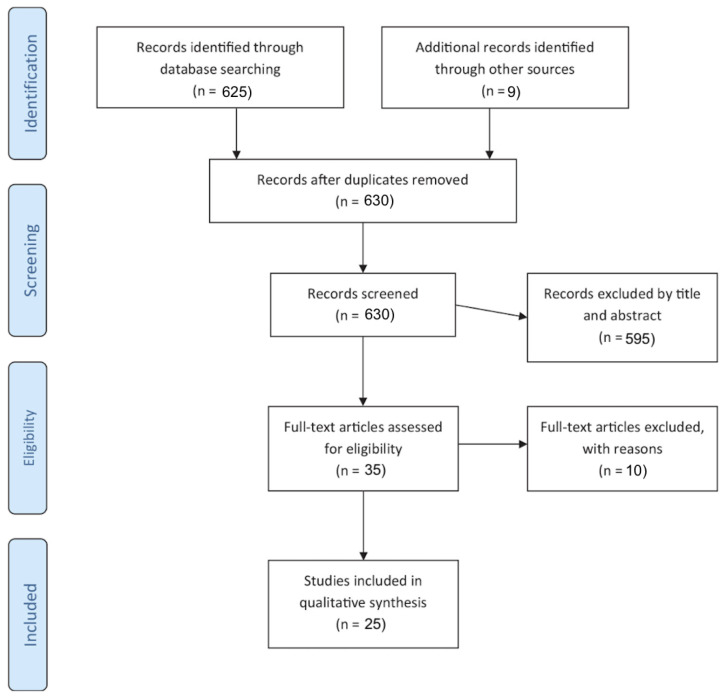
Search flow.

Figure 1 summaries the process of article selection for this review. The search of the PubMed Data base provided a total of 625 citations. Furthermore, nine additional studies which met the inclusion criteria were identified by reviewing the references of selected papers. After adjusting for duplicates, 630 records were screened. Of these, 607 articles were excluded as they were irrelevant to the topic. Upon reviewing the abstracts of articles obtained through a search on Medline (OVID) and SCOPUS, five additional relevant articles were included. After the screening process, 30 studies [17,18,19,20,21,22,23,24,25,26,27,28,29,30,31,32,33,34,35,36,37,38,39,40,41,42,43,44,45,46] assessing NES met the inclusion criteria and were thus included in this scoping review. The need for further research into NES within adult populations is reiterated through the limited number of relevant studies yielded through this search. 

### 3.2. Relevant Studies 

As shown in Table 1, only five studies investigated NES in a representative participant sample [26,27,32,37,44], whilst fourteen studies reported on university students [17,19,24,25,33,34,35,39,40,41,42,43,45,46] and ten studies reported on patients within outpatient clinics [18,20,21,22,23,28,29,30,36,38]. Most studies [17,19,20,21,22,23,24,25,26,28,29,30,32,34,36,37,39,42,43,44,45,46] compared participants who met NES diagnostic criteria with those who did not; however, some studies used no comparison groups [35,40,41], sex [31], age [27] or BMI groups as a comparison [18,38]. 

### 3.3. Diagnostic Criteria 

A variety of tools were used to diagnose NES. including the Other Specified Feeding or eating disorders (OSFED) section of the Diagnostic and Statistical Manual of Mental Disorders, 5th edition (DSM5) [1], Night Eating Questionnaire (NEQ) [47], Night Eating Diagnostic Questionnaire (NEDQ) [48], Eating Disorder Examination Questionnaire (EDE-Q) [49], Night Eating Syndrome History and Inventory (NESHI) [50] and Eating Among Teens Survey (EAT-II) [51]. The discrepancies between the diagnostic criteria used reiterates the need for further research to obtain accurate data regarding the diagnosis and the complications of NES. 

The NEQ can be used as a continuous measure to assess the severity of NES symptoms via a 14-item questionnaire [47]. This questionnaire characterises morning hunger, timing of first food consumption, food craving and control of overeating behaviour before bedtime and during nocturnal awakenings, quantity consumed after dinner, initial insomnia, frequency of nocturnal awakenings and ingestion of food, mood disturbance and awareness of these episodes. Participants in most studies were divided into NES and non-NES groups through a cut-off of 25 or more in the NEQ, which has a positive predictive value (PPV) of 40.7% [47]. Meule et al. [25], however, also utilised a cut-off of 30 or more in the NEQ, increasing the PPV to 72.7% [47]. Additionally, some studies did not include comparison groups and explored the relationship between NEQ results and associated features on a continuum [27,31,33,35,40,41]. 

Additionally, only one study utilised an additional self-reported questionnaire, NESHI, to confirm the diagnosis of NES obtained through the NEQ [29]. The NESHI [50] is a semi-structured interview assessing a typical 24 h food intake, as well as information on average weekly nocturnal ingestions, sleep routine, mood symptoms, weight and diet history, medical history about NES symptoms and previous treatment [52]. Furthermore, three studies utilised the NEDQ as the sole measure to classify diagnostic criteria for NES [17,21,42]. The NEDQ is a 22-item questionnaire which employs a hierarchical scoring method to allocate participants into four categories (normal, mild, moderate and full syndrome) based on the number of symptoms of NES that are present [48]. 

### 3.4. Weight Status 

Twenty-seven studies examined the association between NES and weight status. Findings concerning the relationship between NES and BMI provided inconclusive and contradictory results, 11 studies reported no significant relationship [17,19,21,22,28,29,35,38,39,40,43] and 15 studies reported a statistically significant positive relationship [18,20,23,24,25,27,30,31,32,33,34,36,37,41,42]. One study reported a significant relationship with evening hyperphagia and no relationship with nocturnal ingestions [26].

Additionally, three studies observed other indicators of weight status, including weight (kg) [29], waist-to-hip ratio [38] and waist circumference [31]. Gallant et al. [31] and Kara et al. [38] reported no significant relationship between NES and waist circumference in men and women and waist–hip ratio. In contrast, Kara et al. [38] noted a significant relationship between NES and waist–height ratio. Similarly, Cleator et al. [29] observed a significant positive relation between NES and increased weight.

These inconsistencies may be accounted for by demographic features of participants, which may moderate the association between NES and weight status. Age was noted to moderate the relationship between NES and BMI in one study, which used age as a variable to investigate the impact of NEQ scores on BMI [27]. A significant positive association was reported within participants aged between 31 and 60 years; however, this association was not observed in the other age groups [27]. Consistent with previous studies investigating younger population samples [33,40], six studies [17,19,35,39,40,43] observed no significant relation between NES and BMI when reporting on a sample of college students. In contrast, Kandeger et al. [24], Meule et al. [25] and El Ayoubi et al. [42] reported a significant relationship between NES and BMI when reporting on university students in Turkey, Germany and Lebanon, respectively. Discrepancies between university-aged students and general adult samples [31,37] reiterate the need for further research into NES within adult populations to improve screening and management.

### 3.5. Physical Activity 

Only six studies [17,30,39,43,44,46] explored the impact of NES on physical activity, with four studies [30,39,44,46] reporting a significant association and two studies [17,43] reporting no significant relationship. Levels of physical activity were assessed via the International Physical Activity Questionnaire—Short-form (IPAQ-S) [53], World Health Organization Quality of Life Turkish Short Form (WHOQOL-BREF-TR) [54] and HRQOL scores [6]. Two of the studies which utilised the WHOQOL-BREF-TR and HRQOL did not specifically discuss the physical domains [44,46]. 

Yahia et al. [17] attributed this lack of relationship between physical activity in NES and non-NES to the reduced age of participants (mean age: 20.6 ± 1.68 years). This was reiterated by Hamdan et al. [43], who reported no significant relation between NES and any level of physical activity (sedentary, moderate or high) in a university student sample. This contrasted with Hamurcu et al. [46] and Runfola et al. [39], who reported a significant relation in a population of university students (mean age = 21.4 ± 3.1 and 20.9 ± 1.7 years, respectively), although competitive athletes comprised 59.6% of the sample size for Runfola et al. Lent et al. [30] reported a significant relationship between higher NEQ scores and engaging in moderate-to-vigorous physical activity, although the mean age of participants was older (mean age: 51.1 ± 15 years). Similar results were obtained by Kim et al. when exploring a representative sample of the Korean general population [44]. 

Furthermore, Yahia et al. [17] reported on a small sample size of largely female participants, whilst Runfola et al. [39] reported on a larger sample size (n = 1636) of both men and women. Lent et al. [30], like Yahia et al. [17], reported on a largely female population sample (66.7%), and like Runfola et al. [39], this study utilised a large sample size (n = 1017). Although a larger sample was used in Runfola et al. [39], the majority of participants were athletes, who are not representative of the general population. These inconsistencies highlight the need for further research within large, general-population samples. 

### 3.6. Quality of Sleep 

Thirteen studies explored the impact of NES on quality of sleep [17,20,23,24,26,28,29,30,33,35,36,41,45]. This was primarily achieved through self-reported questionnaires, such as the Pittsburgh Sleep Quality Index (PSQI) [55], Morningness–Eveningness Questionnaire (MEQ) [56], Insomnia severity index (ISI) [57] and Epworth Sleepiness Scale (ESS) [58]. In contrast, Geliebter et al. [21] utilised polysomnography to objectively assess sleep quality via the Apnoea–Hypopnoea Index (AHI) [59] and diagnose obstructive sleep apnoea (OSA). All studies using the PSQI, MEQ and ESS revealed significant relationships between poor sleep quality and NES, whilst the AHI did not. Although objective data were obtained, Geliebter et al. [21] were limited in providing further information addressed through questionnaires, such as sleep duration, daytime sleepiness and impact of quality of life [11]. Moreover, several studies [17,20,28,33,35,41,45] analysed sleep quality over a month using the PSQI, thus increasing their reliability. These findings support the need for further longitudinal research into the interrelation between NES and aspects of sleep to develop effective screening and management of sleep problems which result from NES. 

### 3.7. Medical Conditions 

Five studies [21,28,29,31,36,42,43] investigated the impact of NES on chronic health conditions, including Type 2 diabetes Mellitus (T2DM) [28,29,31,36], metabolic syndrome [31], OSA [21,29] and cardiovascular disease [31,36]. Two additional studies reported on “chronic diseases” and NES, both reporting no significant relationship, although no specifications were reported regarding which conditions [42,43]. 

Of the four studies exploring the relationship between NES and T2DM [28,29,31,36], only Hood et al. [28] reported significant correlation between NEQ scores and HbA1C. In contrast, Cleator et al. [29,30] and Antelmi et al. [36] reported no significant relation between NES and T2DM, as well as other concomitant diseases, including hypertension, cardiovascular disease and thyroid pathologies. Similarly, Gallant et al. [31] identified no significant relation between NEQ in men and women and metabolic syndrome or T2DM when exploring biochemical markers (glucose, HDL cholesterol, triglycerides). Furthermore, only Gallant et al. [31] examined the interrelation between cardiac health, metabolic syndrome and NES, reporting that higher NEQ scores were associated with lower blood pressure in women and a larger waist circumference and higher triglycerides in men. None of the four studies investigating NES and T2DM were conducted on samples representative of the general population; two of the studies [28,29] were conducted within endocrinology outpatient clinics and another was conducted in restless leg syndrome patients from a sleep centre [36]. Furthermore, due to the small sample sizes used in studies examining the association between medical conditions and NES [28,29,31,36], these results may not be clinically significant for the general population.

Two studies [21,29] explored the impact of NES on obstructive sleep apnoea (OSA) using self-reported data on small sample sizes. Both studies reported no significant association between OSA and NES. Additionally, Geliebter et al. [21] did not observe any significant relationship between NES and AHI. Tools to assess OSA varied; Geliebter et al. [21] utilised polysomnography to diagnose OSA, with the threshold criterion of an AHI greater than or equal to 5; Cleator et al. [29], however, did not report how a diagnosis of OSA was made. Further exploration of large samples of participants representative of the general population and clearly defined methods of diagnosing OSA will provide an improved understanding of NES and its relationship with OSA. 

## 4. Discussion

This scoping review investigated the associations between NES and Body Mass Index (BMI; kg/m^2^), physical health and sleep conditions. Thirty studies were identified that reported on associations between physical health problems and NES over the past decade [17,18,19,20,21,22,23,24,25,26,27,28,29,30,31,32,33,34,35,36,37,38,39,40,41,42,43,44,45,46]. Whilst there were inconsistencies in representative and larger-sample studies, NES appeared to be associated with higher BMI, less physical activity and poorer sleep quality. It was not, however, associated with T2DM. Inconsistencies and the lack of association with T2DM may have been because of smaller and less-representative population samples. This review also identified a number of limitations of the extant research. 

### 4.1. Weight Status 

This review found a positive relationship between NES and BMI in 16 of 27 studies [18,20,23,24,25,26,27,30,31,32,33,34,36,37,41,42]. Additionally, three studies observed other indicators of weight status, including weight (kg) [29], waist-to-hip ratio [38] and waist circumference [31], which are regarded as more sensitive universal screening tool than BMI to detect increased metabolic risk [60]. An explanation for the inconsistency in results is the use of different measurement methods. Furthermore, demographic features, most notably age, may have moderated associations [61]. For example, Nolan et al. [33] reported a significant relationship for community participants (mean age: 42.9 ± 0.6 years), but not for college students (mean age:18.7 ± 0.1years). However, Nolan et al. [33] did not further explore age as a moderator. Notably, six studies [17,19,36,39,40,43] reporting on a sample of college students and two studies [33,40] investigating younger population samples found no significant relation between NES and BMI. 

### 4.2. Physical Activity 

Six studies [17,30,39,43,44,46] reported incongruous findings when exploring the relationship between NES and physical activity. This may result from the use of different tools when assessing physical activity, differences in sample sizes and the exploration of different age groups. Of the six studies, only one study utilised a large representative sample of the general population (n = 34,434) [44]. As such, further research into representative populations of adults may glean a better understanding of the interaction between NES and physical activity. 

### 4.3. Quality of Sleep 

Sleep quality was assessed in 13 studies [17,20,23,24,26,28,29,30,33,35,36,41,45]. All studies which utilized self-reported questionnaires reported a significant association between NES and sleep quality, whilst Geliebter et al. [21], who used objective measures, did not. These inconsistencies may result from the use of subjective versus objective measures to assess sleep quality. The NEQ [47], which was the primary tool used to diagnose NES, strongly endorses symptoms related to nocturnal ingestions and sleep (for example, “trouble falling asleep”, “trouble staying asleep” and “waking up at night”), which are also assessed in self-reported questionnaires assessing sleep quality, such as the PSQI [55] and ISI [57]. These results reiterate the need for more objective measures in conjunction with subjective questionaries to assess the association of sleep quality and NES. 

### 4.4. Medical Conditions

This scoping review found four studies [28,29,31,36] which explored the relationship between NES and T2DM, of which only one reported a significant relationship 28. Only Gallant et al. reported on metabolic syndrome, reporting no significant relationship [31]. Neither of the two studies exploring the association between NES and cardiovascular disease reported a significant relationship [31,36]. Both studies investigating the relationship between NES and OSA identified no significant relationship [21,29].

Although the results were largely congruent, the population samples examined within these studies were not representative of a general adult sample. For example, Hood et al. [28] reported on a largely African American sample population (58%), whilst Cleator et al. [29] reported only on Caucasians. Consequently, it is crucial to conduct further research to assess the impact of NES on cardiac and metabolic health within larger representative sample populations. These inconsistencies reiterate the need for further investigation of the impact of NES on glycaemic control and metabolic functioning in patients with T2DM. 

### 4.5. Strengths and Limitations 

Strengths and limitations of this scoping review 

A scoping review was appropriate as we investigated a broad research topic rather than a single research question. Three data base searches were conducted, dating from 2013; whilst this provided a contemporary focus, it is possible that relevant earlier papers and unpublished grey literature research were missed. Two authors (SS and PH) reached consensus on data that were extracted. However, because we performed a scoping review rather than a systematic review, there was no critical appraisal of studies, no application of PICO framework, no estimation of bias in the studies and no meta-analysis performed. 

Strengths and limitations of the extant literature 

A limitation of the extant research is the variety of diagnostic instruments and criteria used to diagnose NES. Some studies utilised only one method to assess NES, while others used a combination of interview, DSM criteria and questionnaires. Twenty-seven studies [18,19,20,22,23,24,25,26,27,28,29,30,31,32,33,34,35,36,37,38,39,40,41,43,44,45,46] utilised the NEQ [47] as the diagnostic criterion for NES and three studies [17,21,42] used the NEDQ [48]. Other measures of NES used in these studies included the EDE-Q [49], NESHI [50] and EAT-II [51]; however, these were used as adjuncts to the NEQ [47] and NEDQ [48].

A further limitation is that most studies were observational rather than experimental, with the majority of the literature reviewed in this scoping review being cross-sectional studies. There was a mixed quality of samples, with five large representative samples [26,27,32,37,44], but many (i.e., 25 studies) smaller and less representative samples. 

## 5. Conclusions

NES is an emerging area for clinical investigation, evaluation and intervention. It is evident through this review that there is a need for further research into NES and its associated features within a representative adult population sample. While few studies have been conducted, data are inconsistent, and thus it is imperative to conduct further research to accurately understand the complex interaction between NES and its associated features. By conducting further research on NES within general population samples, improved diagnostic measures and management plans can be developed to improve the overall health of the community. 

## Figures and Tables

**Table 1:** Relevant studies and their results.

Pub Date, Author	Study Design	Sample Size	Location	Comparison	Participant Features	Age in Years (Mean Age)	Sex	Other Features	Criteria/Questionaries Used	Cut-Off for NES Criteria	Main Results
2017. Yahia N et al. [17]	Case– control	413	USA	Any NES (mild, moderate and full) (n = 51) No NES (n = 362)	Central Michigan University students	18–26 (20.6 ± 1.68 SD)	323 females, 90 males	Self- reported	NEDQ, PSQI, IPAQ-S	Categorised via NEDQ	Relation between NES, sleep duration (*p* = 0.023) and higher PSQI score (*p* = 0.007). Relation between NES and BMI, eating habits, physical activity and smoking status (NS).
2021. Sutcu C et al. [18]	Cross- sectional	420	Turkey	Normal weight: BMI 18.50–24.99 (n = 105) Overweight: BMI 25–29.99 (n = 105) Obese: BMI 30–39.99 (n = 105) Morbidly obese: BMI > 40 (n = 105)	Endocrinology outpatient clinic	18–65 (42 ± 12 SD)	288 females, 132 males	Face-to-Face interview	NEQ, BMI, waist circumference	≥25 on NEQ	Relation between NES and waist circumference (*p* < 0.05). Relation between NES and morbid obesity (*p* < 0.001).
2020. Riccobono G et al. [19]	Cross-sectional	1136	Italy	NES (n = 60) No NES (n = 1076)	Italian university students	MD (25.97 ± 10.78 SD)	774 females, 360 males	Self-reported	NEQ, MEQ, BMI, SPAQ	≥25 on NEQ	Relation between NEQ and MEQ scores are significantly inversely correlated (*p* < 0.01). Relation between BMI and NES (NS).
2014. Kucukgoncu S et al. [20]	Cross-sectional	155	Turkey	NES (n = 33) No NES (n = 122)	Patients with depression in outpatient clinic	18–60 (35.80 ± 8.78 SD)	125 females, 30 males	Clinical interviews	NEQ, structural clinical interview for DSM-IV axis I diagnosis, PSQI	≥25 on NEQ	Relation between NES and Global PSQI scores (*p* < 0.001). Relation between NES and BMI (*p* = 0.041).
2016. Geliebter A et al. [21]	Cross-sectional	84	USA	NES/subthreshold NES (n = 30) No NES (n = 54)	Pts referred for poly-somnography at the Sleep Disorders Institute	18–81 (43.2 ± 13.3 SD)	34 females, 50 males	Self-reported	NEDQ, BMI, AHI	Categorised via NEDQ	Relation between NES and BMI, OSA or AHI (NS).
2015. Saraçlı Ö et al. [22]	Cross-sectional	1188	Turkey	NES (n = 97) No NES (n = 336)	Psychiatric out-patients	18 + (37.75 ± 12.02 SD)	777 females, 411 males	Clinical interview	DSM-iV, NEQ, BMI	≥25 on NEQ	Relation between NES and BMI (NS).
2017. Dorflinger LM et al. [23]	Cross-sectional	110	USA	NES (n = 12) No NES (n = 98)	MOVE! weight management program, veterans	MD (61.6 ± 8.5 SD)	11 females, 99 males	Self-reported	NEQ, ISI, BMI	≥25 on NEQ	Relation between NEQ score and ISI (*p* < 0.001). Relation between NEQ score and higher BMI (*p* < 0.05).
2018. Kandeger A et al. [24]	Cross-sectional	383	Turkey	NES (n = 20) No NES (n = 363)	University students	17–37 (21.1 ± 0.1 SD)	230 females, 153 males	Self-reported	NEQ, MEQ, ISI, BMI	≥25 on NEQ	Relation between NES and BMI (*p* < 0.01). Relation between NES and ISI scores (*p* < 0.001).
2014. Meule A et al. [25]	Cross-sectional	729	Germany	NES (n = 11) No NES (n = 718) NEQ > 25 (n = 9) NEQ > 30 (n = 2)	University students	18–47 (23.55 ± 3.89 SD)	561 females, 168 males	Online self-reported	NEQ, MES, BMI	≥25 on NEQ	Relation between NES and BMI (*p* < 0.01).
2021. Matsui K et al. [26]	Cross-sectional	8348	Japan	No NES (n = 8024) Nocturnal ingestions (n = 208) Evening hyperphagia (n = 119)	General Japanese population	16–79 (MD)	4182 females, 4166 males	Online self-reported	NEQ, BMI, ISI	≥25 on NEQ	Relation between evening hyperphagia and BMI (*p* < 0.05), average sleep duration of < 6 h (*p* < 0.001), later sleep–wake schedule (*p* < 0.001), ISI score of 8–14 points (*p* < 0.05), and ISI score of 15–28 points (*p* < 0.001). Relation between nocturnal ingestions and earlier sleep–wake schedule (*p* < 0.001), ISI score of 8–14 points (*p* < 0.001) and ISI score of 15–28 points (*p* < 0.001). Relation between nocturnal ingestions and BMI (NS).
2014. Meule A et al. [27]	Cross-sectional	2317	Germany	21–30 years (n = 332) 31–40 years (n = 335) 41–50 years (n = 450) 51–60 years (n = 437) 61–70 years (n = 399) > 70 years (n = 364)	Representative sample of German adults	21–92 (51.45 ± 16.97 SD)	1245 females, 1072 males	Self-reported	NEQ, BMI	No cut-off	Weak positive relation between BMI and NES (*p* < 0.001).
2014. Hood MM et al. [28]	Cross-sectional	194	USA	NES (n = 13) No NES (n = 181)	Endocrinology clinic outpatients with T2DM	18–65 (58.4 ± 13.0 SD)	135 females, 59 males	Self-reported	DSM5, NEQ, PSQI, ESS, MEQ, BMI	≥25 on NEQ	Relation between NES and poorer sleep quality (*p* < 0.001), more daytime sleepiness (*p* = 0.002) and shorter sleep duration (*p* = 0.009). Relation between NEQ scores and HbA1C (*p* = 0.2). Relation between NES and BMI, age (NS).
2014. Cleator J et al. [29]	Cross-sectional	81	UK	NES (n = 31) No NES (n = 50)	UK outpatient clinic, all Caucasian	18–68 (44.6 ± 11.6 SD)	46 females, 35 males	Self-reported	NEQ, NESHI, weight, BMI, comorbidities, sleep	≥25 on NEQ	Relation between NES and weight (*p* = 0.04). Relation between NES and BMI, T2DM, OSA and sleep duration (NS).
2022. Lent MR et al. [30]	Cross-sectional	1017	USA	NES (n = 48) No NES (n = 969)	General internal medicine, primary care, or weight management clinics	18 + (51.1 ± 15.0 SD)	790 females, 227 males	Self-reported online	NEQ, BMI, frequency of naps (<1/wk, 2–3/wk or 4+/wk), MCTQ, IPAQ-SF	≥25 on NEQ	Relation between NES and higher BMI (*p* < 0.001), shorter sleep duration (*p* < 0.001), napping < two times per week (*p* = 0.002) and engaging in moderate-to-high physical activity (*p* = 0.005).
2014. Gallant A et al. [31]	Longitudinal cohort study	615	Canada	Women (n = 310) Men (n = 305)	Adults enrolled in QUALITY (Quebec Adiposity and Lifestyle Investigation in Youth)	18+ Females (40.3 ± 5.1 SD) Males (42.5 ± 5.9 SD)	310 females, 305 males	Self-reported	NEQ, BMI, waist circumference, weight, ATP III criteria, bloods (BGL, lipids), BP	≥25 on NEQ	Relation between BMI and NEQ in women (*p* < 0.001) and in men (*p* = 0.04). Relation between higher NEQ and low BP in women (*p* < 0.05) and BP in men (NS). Relation between NEQ in men and larger waist circumference (*p* < 0.05) and increased triglycerides (*p* < 0.01). Relation between NEQ in women and larger waist circumference and increased triglycerides (NS). Relation between NEQ in men and women and metabolic syndrome or T2DM (NS).
2014. de Zwaan M et al. [32]	Cross-sectional	2456	German	NES (n = 27) No NES (n = 2432)	Representative sample of the German general population	14–92 (48.1 ± 19.0 SD)	1256 females, 1200 males	Self-reported	NEQ, BMI	≥25 on NEQ	Relation between NES and BMI (*p* = 0.018).
2017. Nolan LJ et al. [33]	Cross-sectional	722	USA	Students (n = 254), community members (n = 468)	University students and community member	25 + Student group (18.7 ± 0.1 SD) Community group (42.9 ± 0.6 SD)	421 females, 301 males	Online self-reported	NEQ, NEDQ, BMI, PSQI	≥25 on NEQ	Relation between NES and BMI (*p* < 0.001). Relation between NES and PSQI (*p* = 0.006).
2014. Meule A et al. [34]	Cross-sectional	305	German	NES (n = 4, 1.24%) No NES (n = 301)	University students	18–47 (23.55 ± 3.89 SD)	MD	Online self-reported	NEQ, MES, r-MEQ,	≥25 on NEQ	Relation between NES and BMI and BMI (*p* < 0.001).
2017. Aloi M et al. [35]	Cross-sectional	444	Italy	No comparison group	University students	18+ (21.4 ± 2.3 SD)	327 females and 247 males	Self-reported	NEQ, EDE-Q, PSQI, BMI	≥25 on NEQ	Relation between NEQ and BMI (NS). Relation between NEQ and age (NS). Relation between NEQ and PSQI (*p* < 0.001).
2014. Antelmi E et al. [36]	Cross-sectional	120	Italy	NES (n = 20) No NES (n = 100)	Resting leg syndrome in patients	18+ (63.8 ± 11.5 SD)	83 females, 37 males	Telephone	NEQ, ESS, BMI	≥25 on NEQ	Relation between NES and BMI was significantly higher in RLS patients (*p* = 0.023). Relation between NES and insomnia complaints and ESS (NS). Relation between NES and concomitant disease (HTN, CVD, DM, etc.) (NS).
2018. Olejniczak D et al. [37]	Cross-sectional	611	Poland	NEQ ≥25 (n = 12) NEQ ≥30 (n = 4) No NES (n = 595)	General population	19–30 (22.7)	611 females, 0 males	Self-reported	NEQ, BMI	≥25 on NEQ	Relation between NES and higher BMI (*p* = 0.022).
2020. Kara Y et al. [38]	Case-control	421	Turkey	NES (n = 92) No NES (n = 329) class I obesity (n = 150) class II obesity (n = 141) class III obesity (n = 130)	Obesity outpatient clinic	18+ Class I (49.49 ± 12.49) Class II (48.43 ± 11.81) Class III (49.05 ± 11.40)	349 females, 72 males	Self-reported	NEQ, BMI, waist and hip circumference	≥18 on NEQ	Relation between NES and BMI and waist–hip ratio (NS).
2014. Runfola CD et al. [39]	Cross-sectional	1636	USA	NES (n = 67) No NES (n = 1569)	University students, athletes	18–26 (20.9 ± 1.7 SD)	972 females, 664 males	Self-reported	NEQ, EDE-Q, EAT-II, HRQOL, BMI	≥25 on NEQ	Relation between BMI and NES (NS). Relation between NES and lower HRQOL (*p* < 0.001).
2018. He J et al. [40]	Cross-sectional	1237	China	No comparison group	University students	18+ (19.96 ± 1.36 SD)	670 females, 567 males	Self-reported	NEQ, BMI, EDI	No cut-off	Relation between BMI and NEQ (NS).
2014. Yeh SS et al. [41]	Cross-sectional	330	Australia	No comparison group	College students (48.4%), university staff, friends, and colleagues	18–87 (27.42 ± 10.36 SD)	223 females, 107 males	Self-reported	NEQ, PSQI, BMI	≥25 on NEQ	Relation between NES and BMI (*p* < 0.01) and reduced sleep duration (*p* < 0.01).
2022. El Ayoubi LM et al. [42]	Cross-sectional	404	Lebanon	No NES (n = 239) Mild NES (n = 75) Moderate NES (n = 59) Full NES (n = 31)	University students, 72% female		291 females, 113 males	Self-reported online	NEDQ, GHQ-12, BMI	Categorised via NEDQ	NEDQ and BMI (*p* < 0.0001). NEDQ and GHQ (*p* < 0.0001).
2023. Hamdan M et al. [43]	Cross-sectional	475	Palestine	NES (n = 141) No NES (n = 334)	University students	18–25 (19.8 ± 1.4 SD)	253 females, 197 males	Self-reported	NEQ, BMI, SF-IPAQ, Medical profile (Chronic diseases and duration)	≥25 on NEQ	NES and BMI (NS). NES and medical history (NS). NES and physical activity (NS). NES and chronic disease (NS).
2023. Kim W et al. [44]	Cross-sectional	34434	Korea	NES (n = 197) No NES (n = 344144)	Representative sample of the Korean general population	19 + (MD)	17729 females, 16705 males,	2019 Korea Community Health Survey (KCHS)	NEQ, 3-level EuroQoL-5 Dimension Index (EQ-5D-3L)	≥25 on NEQ	NES and lower HRQOL (*p* < 0.001).
2022. Suna G et al. [45]	Cross-sectional	568	Turkey	NES (n = 24) No NES (n = 544)	University students	18–25 (20.32 ± 1.61 SD)	447 females, 121 males	Self-reported	PSQI, NEQ, BMI, WHR	≥25 on NEQ	NES and higher PSQI (*p* = 0.001).
2022. Hamurcu P. [46]	Cross-sectional	846	Turkey	No NES (n = 273) NES (n = 573)	University students	MD (21.4 ± 3.1 SD)	712 females, 134 males	Self-reported online	NEQ, PSQI, World Health Organization Quality of Life Short Form (WHOQOL-BREF-TR)	≥25 on NEQ	NES and WHOQOL-BREF-TR (*p* < 0.001).

Note: MD (missing data).

## Data Availability

Not applicable.
